# Decoding the Gene Variants of Two Native Probiotic *Lactiplantibacillus plantarum* Strains through Whole-Genome Resequencing: Insights into Bacterial Adaptability to Stressors and Antimicrobial Strength

**DOI:** 10.3390/genes13030443

**Published:** 2022-02-28

**Authors:** Gabriela N. Tenea

**Affiliations:** Biofood and Nutraceutics Research and Development Group, Faculty of Engineering in Agricultural and Environmental Sciences, Technical University of the North, Av. 17 de Julio s-21 Barrio El Olivo, Ibarra 100150, Ecuador; gntenea@utn.edu.ec

**Keywords:** genomic variation, gene variants, SNPs, *Lactiplantibacillus plantarum*, next-generation sequencing, pan-genome, probiotics

## Abstract

In this study, whole-genome resequencing of two native probiotic *Lactiplantibacillus plantarum* strains—UTNGt21A and UTNGt2—was assessed in order to identify variants and perform annotation of genes involved in bacterial adaptability to different stressors, as well as their antimicrobial strength. A total of 21,906 single-nucleotide polymorphisms (SNPs) were detected in UTNGt21A, while 17,610 were disclosed in the UTNGt2 genome. The comparative genomic analysis revealed a greater number of deletions, transversions, and transitions within the UTNGt21A genome, while a small difference in the number of insertions was detected between the strains. A divergent number of types of variant annotations were detected in both strains, and categorized in terms of low, moderate, and high modifier impact on the protein effectiveness. Although both native strains shared common specific genes involved in the stress response to the gastrointestinal environment, which may qualify as a putative probiotic (bile salt, acid, temperature, osmotic stress), they were different in their antimicrobial gene cluster organization, with UTNGt21A displaying a complex bacteriocin gene arrangement and dissimilar gene variants that might alter their defense mechanisms and overall inhibitory capacity. The genome comparison revealed 34 and 9 genomic islands (GIs) in the UTNGt21A and UTNGt2 genomes, respectively, with the overrepresentation of genes involved in defense mechanisms and carbohydrate utilization. In addition, pan-genome analysis disclosed the presence of various strain-specific genes (shell genes), suggesting a high genome variation between strains. This genome analysis illustrates that the bacteriocin signature and gene variants reflect a niche-inherent pattern. These extensive genomic datasets will guide us to understand the potential benefits of the native strains and their utility in the food or pharmaceutical sectors.

## 1. Introduction

Bioprospecting tropical plants to search for beneficial endophytic microorganisms that produce novel biotechnological molecules remains of interest [1,2]. Microorganisms associated with plants are subjected to constant metabolic and environmental interactions; however, the diversity of the produced molecules is linked to the host chemical composition [3,4]. 

The genome-scale analysis of beneficial bacteria such as lactic acid bacteria (LAB) represents a fundamental approach to investigating their physiological performance or predicting their putative probiotic capabilities, adaptability to different environments (tolerance to bile salt, acids, temperature, osmotic stress, etc.), and post-/metabiotic features (production of antimicrobials, secondary metabolites, enzymes, exopolysaccharides, etc.). Douillard et al. [5] performed a comparative genome analysis of two commercialized *Lactobacillus casei* strains isolated from different fermented products, showing a limited number of SNPs in the genome, among other features that implied the high similarity among these strains. Moreover, Botta et al. [6] studied the genomes of three *L. plantarum* strains, indicating that the high flexibility and metabolic versatility of these strains—which can acquire, substitute, or delete genomic regions—are ligated to their distinct environmental niches of origin. In another study, three *Lactococcus lactis* isolates showed few mutations within various genes involved in amino acid production/transport and the mismatch repair mechanism (*mut*L gene), highlighting that the high frequency of mutations in this region might be responsible for the adaptation of strains from plant to dairy environments [7]. Among probiotic *Lactobacillus*, the most documented strain is *Lactobacillus rhamnosus* GG (ATCC 53103) [8], isolated from the intestinal tract of a healthy human. Additionally, *L. plantarum* WCFS1, isolated from human saliva, has been used as a reference strain for many genomic studies [9,10]. Previous research indicates that *L. plantarum* strains isolated from various niches exhibit high genetic variation and phylogenetic patterns [11]. In addition, no direct genetic connection between genomic characteristics and host niches was found in the pan-genome analysis [12]. More recent genomic comparison between several *L. plantarum* strains originating from pickles, fermented sauce, and human feces indicated that the genetic variation of LAB strains is associated with the host niche [13]. However, the capacity of microbes to adapt to different niches depends on the genetic repertoire and the capacity of these strains to counteract externally exerted physicochemical challenges [14]. Therefore, the microbial composition in these environments is irregular, and depends on intrinsic (i.e., physical and nutritional conditions) and extrinsic (i.e., environmental and harvesting conditions) parameters of the plant matrix [15]. Although several species have been identified, the selection of new strains with valuable biotechnological properties remains a topic of interest [16].

Native tropical fruits consumed in Ecuador are likely to be an excellent microenvironment to search for such useful microorganisms [17]. These microorganisms produce antimicrobials that enable their survival in competitive habitats with other microorganisms, protecting the host against other bacterial or fungal pathogens [18]. Native lactobacilli from wild fruits regularly face extreme variation in conditions such as temperature, nutrients, and pH; however, their metabolic capacities—including antimicrobial properties—are more competitive than those of other microorganisms isolated from other niches [2]. The biochemical versatility and diversity of these microorganisms suggest that many actively produced molecules remain unknown. Therefore, these probiotics can be an alternative to conventional antibiotics or other therapeutic drugs. 

Previously, we prospected the microbiota of several wild fruits of the Ecuadorian Amazon [19] to select potential probiotic and antimicrobial LAB producers. Among these unique biological niches, two strains of *Lactiplantibacillus plantarum*—UTNGt21A and UTNGt2—were selected, and their genomes were characterized. Although they belong to the same species and share conserved genes responsible for the biosynthesis or degradation of structural compounds such as proteins, lipids, and DNA, they are highly divergent, as demonstrated by the differences in their antimicrobial gene cluster organization (i.e., bacteriocins and non-ribosomal peptide biosynthesis gene clusters), as well as their diversity of bioactive molecules and secondary metabolites. In this study, the whole-genome resequencing of both native strains was carried out in order to identify gene variants and discern metabolic features and genes linked to the adaptability of bacteria to different environments. Moreover, the identification of GIs and insertion sequences (ISs) might explain the adaptability, metabolic versatility, and fitness of both native strains. Furthermore, pan-genome analysis was conducted in order to detect strain-specific genes (shell genes). The use of NGS for whole-genome sequencing (WGS) and gene annotation, followed by the identification of the type of SNPs, indels, transitions, and transversions, along with the in-depth evaluation of the antimicrobial cluster gene variants of the target strains and of the reference counterpart (*L. plantarum* WCFS1), might help to understand their genetic variation, genomic complexity, adaptation to different niches, and overall antimicrobial capacity. 

## 2. Materials and Methods

### 2.1. Bacterial Strains 

*Lactiplantibacillus plantarum* UTNGt21A and UTNGt2 strains were isolated from wild fruits of *Solanum quitoense* Lam. (naranjilla) and *Theobroma grandiflorum* (white cacao), following the procedure described in [19]. The genome assembly data of the UTNGt2 and UTNUTNGt21A strains were previously deposited to the NCBI database under the BioProject PRIJNA705232 with BioSample SAMN18053630 on 26 February 2021, and BioProject PRIJNA740042 with BioSample SAMN19816459 on June 23, 2021. The assembly of the *Lactobacillus plantarum* WCFS1 strain is available in GenBank (https://www.ncbi.nlm.nih.gov/assembly/GCF_000203855.3, accessed on 5 September 2020), providing the basis for data analysis of the whole-genome sequencing (reference sequence). 

### 2.2. Whole-Genome Sequencing, Gene Prediction, and Functional Annotation

The Illumina HiSeq X Ten platform was used for sequencing using a custom assay by the design service (Macrogen Inc., Seoul, Korea). The detailed construction procedure, sequencing library, cluster generation, quality control, and statistical analyses summarized the basic characteristics of the read contig, and assembly was as described in previous studies [19,20]. To map the reads obtained from the sequencing, the Burrows–Wheeler aligner (BWA) (v0.7.17) and Burrows–Wheeler transform (BWT) (http://bio-bwa.sourceforge.net/, accessed on 11 September 2020) algorithms were used [21]. The gene prediction and functional annotation were performed as previously described in [19].

### 2.3. Genome and Pan-Genome Comparison Analysis

The circular and linear genome comparison diagram was predicted using CGView [22]. Multiple genome alignment under Mauve (with default settings: the value of minimal Locally Collinear Blocks (LCBs) was equal to 1000; island size: 50; backbone size: 50; maximum gap: 50) was used to perform the synteny analysis [23]. The “Mauve Contig Mover” (default settings) was used to order a draft genome of UTNGt21A and UTNGt2 relative to a related reference genome. Pan-genome analysis was carried out by using Roary v1.007001 [24] with the MAFFT v7.427 aligner [25]. Genomic sequences from the three samples were used to cluster the genes encoding complete protein sequences into core (hardcore and softcore) and accessory (shell and cloud) genomes.

### 2.4. Identification of Genomic Islands (GIs) and Insertion Sequences (ISs) within the Genomes of Native Strains

The webserver IslandViewer 4, used to predict GIs, was employed using WCFS1 as a reference strain [26]. Moreover, to search for ISs, the ISfinder tool (ISsaga V.2.0) was used [27]. 

### 2.5. SNPs and Indel Discovery, Transition and Transversion Information, and Variant Annotation 

Produced mass sequence data were used to search for genetic variation. During mapping, duplicated reads can falsely cause erroneous data to stand out. To prevent this, Sambamba v0.6.7 (http://lomereiter.github.io/sambamba/, accessed on 11 September 2020) was used to remove duplicated reads that were identified using mapping information such as start position and CIGAR string (Compact Idiosyncratic Gapped Alignment Report) [28]. After removing duplicates and identifying variants with SAMtools (http://samtools.sourceforge.net/, accessed on 11 September 2020) [29,30], the information of each variant was gathered and classified by chromosomes or scaffolds. The numbers of transitions (Ts) and transversions (Tv), along with the Ts/Tv ratio, were calculated using the base change count. Base changes (DNA substitutions) are of two types: interchanges of purines (A <-> G) or pyrimidine (C <-> T) are transitions, while interchanges of a purine for pyrimidine bases, and vice versa, are transversions. To determine the annotation information, such as amino acid changes of variants, SnpEff v4.3t (http://snpeff.sourceforge.net/, accessed on 11 September 2020) was used [31]. SnpEff generates the genes and transcripts affected by the variant, the location of the variants, and how the variant affects the protein synthesis (e.g., generating a stop codon). Because genes usually have multiple transcripts, a single variant can have different effects on different transcripts. Thus, the impact categories must be used with care, as they were created only to help and simplify the filtering process. A representative transcript was chosen by the gene name obtained from the variant calling analysis. Other transcripts were chosen by the information of neighboring genes that were close enough. 

### 2.6. Detection of Biosynthetic Gene Clusters (BGCs) and Genes Involved in the Adaptability to Several Stressors

The detection of biosynthetic gene clusters was investigated using the BAGEL4 (http://bagel.molgenrug.nl/, accessed on 4 October 2021) webserver [32]. The genes related to the adaptability to pH, bile salt hydrolase, temperature, and osmosis were retrieved from EggNOG annotation results. Moreover, comparison of gene variants was performed using the gene annotations obtained in Section 2.5. 

## 3. Results and Discussion

### 3.1. Comparative Genome and Pan-Genome Analysis Reveals the High Genetic and Niche-Specific Variation of Native Strains

**Figure 1:**
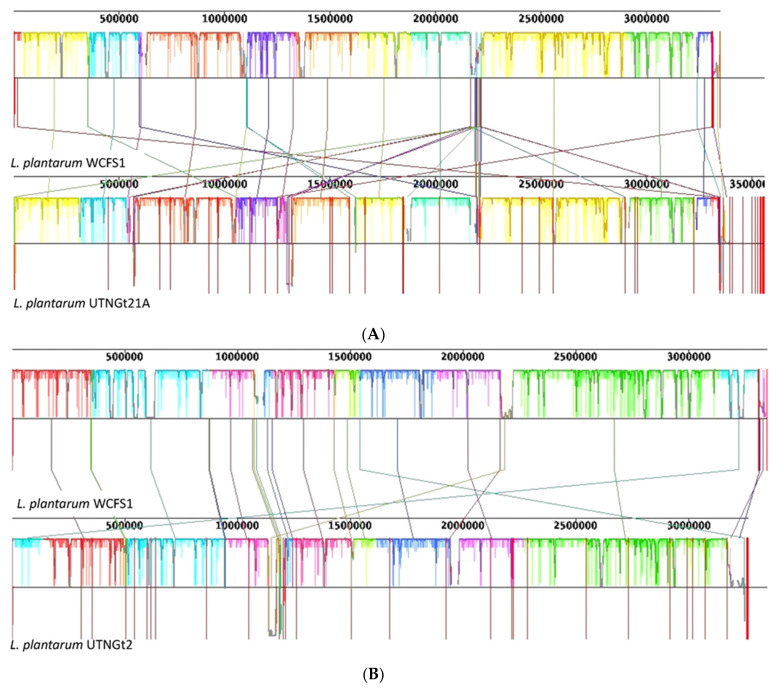
Whole-genome alignments performed using the Mauve contig mover module. The genome of reference strain WCFS1 was aligned with the (**A**) UTNGt21A and (**B**) UTNGt2 draft genomes. White areas indicate low-identity regions between strains. Regions with the same color indicate high-similarity syntenic blocks, and are connected by the same color bars. Red bars indicate boundaries of the original contigs. The numbers above the alignments indicate the nucleotide positions in the WCFS1 genome.

**Figure 2:**
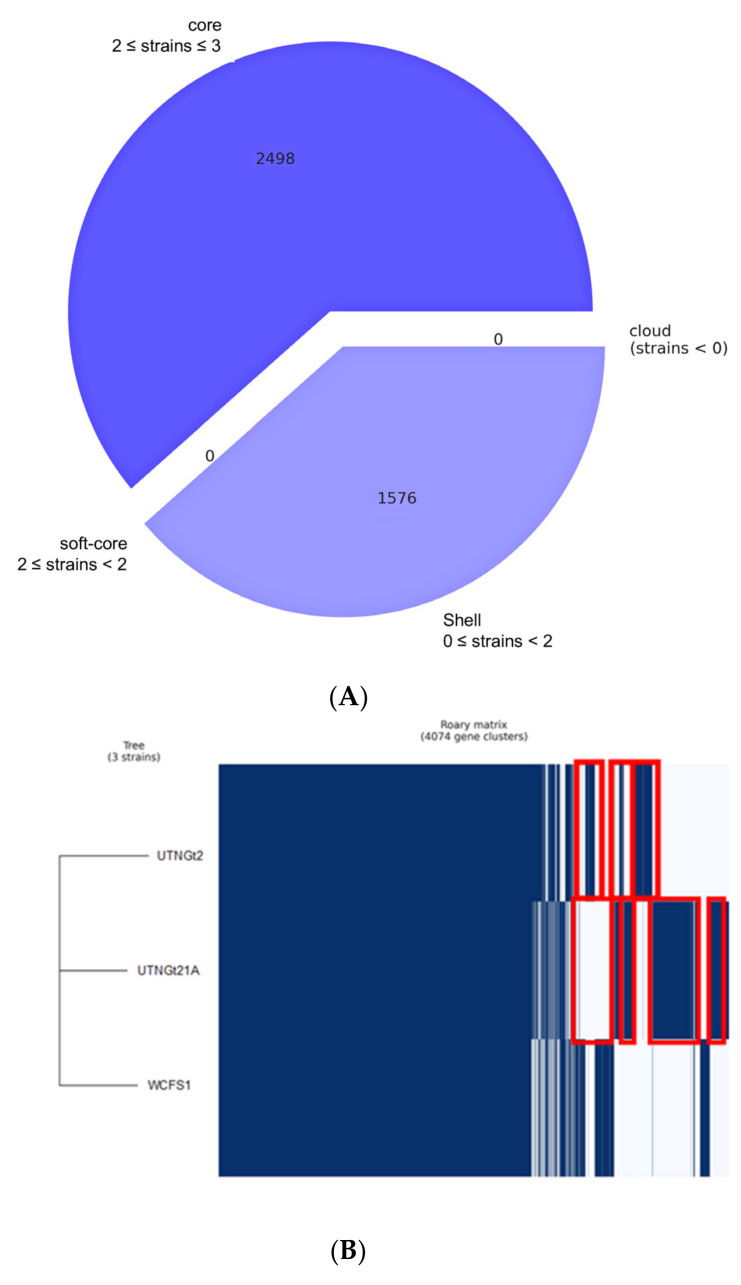
Gene content comparison of the three L. plantarum strains: (**A**) The pie chart shows the number of genes belonging to the core, the soft core, the shell, or the cloud. (**B**) The Roary matrix shows genes typical of each strain and those conserved in all strains. The red rectangular frame indicates several distinct cluster genes of the UTNGT21A and UTNGt2 strains.

To map the reads obtained from sequencing, *Lactobacillus plantarum* WCFS1 was used as a reference genome. For UTNGt21A, 13,121,820 total reads were mapped with 43.12 GC% and Q30 of 94.25%, while for UTNGt2, 11,733,026 total reads were mapped with 43.78 GC% and Q30 of 93.99%. The number of mapped sites, mapping coverage, total number of reads, number of mapped reads, overall mapping ratio, number of mapped bases, and the average alignment depth are shown in Table 1. A circular and linear map comparison is depicted in Figure S1, the UTNGt21A genome being larger than the reference WCFS1 and UTNGt2 genomes. Whole-genome alignments performed using the Mauve contig mover module indicated more rearrangements for the UTNGt21A strain (Figure 1A) than for the UTNGt2 (Figure 1B) strain when both genomes were compared with the reference WCFS1 genome. Although overall high synteny conservation was observed among the three strains, indicating that *L. plantarum* has a very stable genomic structure, numerous gene rearrangements were detected, with highly variable regions located between 1000 kb and 2500 kb (Figure 1A,B). The intersection and connection lines indicate the presence of some regions without LCB outlines, suggesting the presence of strain-specific regions; these differences might be related to the differences in the lifestyle islands (Table S1). These events can occur during evolution or horizontal gene transfer (HGT), indicating the plasticity of the *L. plantarum* genome [33]. Moreover, a total of 4074 genes were subjected to the pan-genome analysis (Figure 2A). The results indicated that the three genomes shared ~2498 common genes, while 1576 genes were sample-specific genes (shell genes). Several gene clusters were distinguished in both native strains (Figure 2B). From the gene presence/absence comparison, several strain-specific proteins involved in the defense mechanism were detected. Among them, a nisin leader peptide-processing serine protease NisP (*nisP*, locus tag: UTNGt21A_02824) indicated 100% sequence identity with peptidase S8 specific protease domain—a lantibiotic (lanthionine-containing antibiotics)-specific protease very similar in structure to serine proteases from several *L. plantarum* strains and *Bacillus* spp. Likewise, two hypothetical proteins of the lanthionine synthetase C family (COG4403) were detected, with 100% identity with the DUF4135-domain-containing protein found in bacterial species and archaea, of ~380 amino acids in length and unknown function. Moreover, a hypothetical plantaricin-C-like protein (locus tag: UTNGt21A_02827) showed 100% identity with a plantaricin-C-like precursor from different *L. plantarum* and an autolytic lysozyme (Lys_2) that was annotated in the UTNGt21A genome. BLASTN protein analysis indicated a specific hit with the bacterial SH3 domain and a non-specific hit with GH25_Lys2-like—a cell wall endolysin produced by *L. fermentum*. This peptide degrades bacterial cell walls by catalyzing the hydrolysis of 1,4-beta-linkages between N-acetylmuramic acid and N-acetyl-D-glucosamine residues [34]. Likewise, CRISPR-associated endonucleases Cas1, Cas2, and Cas9 were annotated in the UTNGt2 genome, but not in the reference WCFS1 and UTNGt21A genomes. BLASTN protein analysis of CRISPR-associated endoribonucleases Cas2 and Cas1 indicated 100% identity with the type-II-A-CRISPR protein Csn2 and CRISPR-associated endonuclease Cas2, respectively, of many *L. plantarum* strains. The presence of these endonucleases might enhance the strains’ stability and adaptation to new niches [35]. Two genes encoding for an arsenical resistance operon repressor (*arsR)* and arsenate reductase (*arsC*) were annotated in all target genomes, while the genes *arsD, arsA*, and *arsB* encoding for the arsenite efflux transporter metallochaperone ArsD, arsenical pump-driving ATPase, and arsenical efflux pump membrane protein ArsB, respectively, were detected in the WCFS1 genome. These results are consistent with previous gene analysis indicating that WCFS1 harbors a protein system involved in arsenic detoxification [36]. Moreover, genes encoding for the biosynthesis of riboflavin synthase (*ribE*) and riboflavin biosynthesis protein RibBA (*ribBA*) were detected in all genomes, while riboflavin biosynthesis protein RibD (*ribD*) and riboflavin transporter RipZ (*ripZ_2*) were annotated in the genomes of both native strains. The gene encoding for riboflavin transporter RibU (*ribU*) was annotated in the UTNGt21A genome. Early studies indicated that several *Lactobacillus* strains harbor the operon for riboflavin biosynthesis [37]. Moreover, we found that the gene encoding for glutamate decarboxylase (*gadB*) was common to all strains, while the gene encoding for aspartate-1 decarboxylase (*panD*) was detected in the UTNGt21A and WCFS1 genomes, but not UTNGt2. Although from gene analysis we cannot predict the impact of these genes on the production of biogenic amines (toxins that, when accumulated in food during storage, might cause human health problems) by the LAB strains, previous in vitro analysis indicated that these decarboxylase enzymes are not sufficient to produce these substances [38]. However, the pan-genome results indicated a high genomic variation among the native strains, which might be related to the addition or deletion of the genes during the adaptation of the species to the fruit microenvironment. Further transcriptomic and proteomic analysis will elucidate the physiological importance of these genes in bacterial survival, pathogen exclusion, and adaptation to different environments. 

### 3.2. Differences between the GIs and ISs Might Explain the Strains’ Adaptability to Different Niches

**Figure 3:**
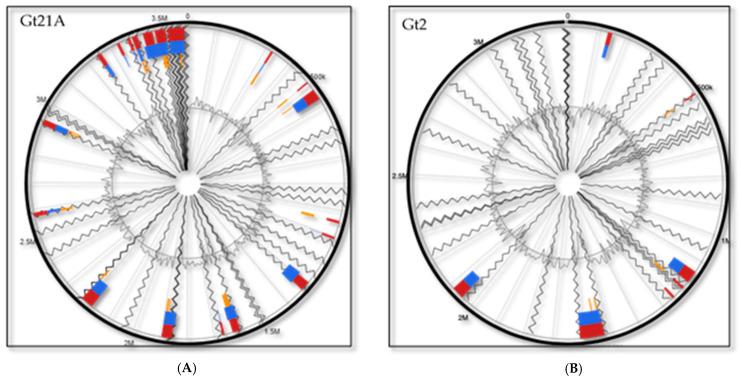
Circular plots visualizing genome islands predicted in the (**A**) UTNGt21A and (**B**) UTNGt2 genomes, aligned against the complete reference genome L. plantarum WCFS1, with blocks colored according to the prediction method. IslandPath-DIMOB (blue), SIGI-HMM (orange), and the integrated results (dark red).

Comparative genomic analysis of native *L. plantarum* strains allows for the identification of niche- or lifestyle-specific genome characteristics. However, acquisition of the host bacterial genomes of bacteriophages, transposons, and other mobile elements via HGT results in the formation of genomic islands (GIs), which might confer fitness benefits on the native strains in specific habits [39]. Genomic analysis via the IslandViewer 4 web tool revealed that the UTNGt21A genome harbored the largest number of islands (34), with a total length of 549074bp, while UTNGt2 harbored 9 GIs with a total length of 214,624bp (Figure 3). In this study, most GIs encoded several hypothetical proteins and species-specific proteins. Thus, within the UTNGt21A genome, several GIs encoding proteins involved in defense mechanisms such as lactococcin-G-processing and transport ATP-binding protein (LagD) and nisin leader peptide-processing serine protease (NisP) were detected (Table S1). Like the results from the pan-genome analysis, GIs identified sequences matching an autolytic lysozyme (lys_2) and two caseinolytic protease-encoding genes (*ClpP1_1*, *Clp1_2*). These bacteriocin-specific genes might enhance the adaptability and competitiveness of the microbe within a niche and, thus, might contribute to the general environmental adaptation of the strain. Within the UTNGt2 genome, GIs encode several proteins involved in the carbohydrate transport PTS system component (e.g., sorbose-specific EIIA, EIIB, EIIC, EIID), PTS system galactitol-specific EIIB component, PTS system N,N′-diacetylchitobiose-specific EIIC component, and fructose-1,6-bisphosphatase class 3. A relatively high number of genes related to carbohydrate utilization, known as “lifestyle adaptation islands”, were previously found in several *L. plantarum* strains [40]; likewise, several hypothetical proteins were found in the GIs of the UTNGt2 strain. In addition, a putative transposon (Tn552 DNA-invertase bin3) and a serine recombinase (PinR) were detected in the GIs of the UTNGt2 genome, while an SPBc2-prophage-derived glycosyltransferase (SunS) was detected within the UTNGt21A genome. No virulence factors or antibiotic-resistance genes were annotated within the GIs. The presence of the PTS carbohydrate system in the UTNGt2 genome might explain the adaptation of the cells to various food matrices. Recent in vitro complementary analysis indicated that the UTNGt2 strain adapted and grew in both milk- and fruit-based matrices, while the UTNGt21A strain adapted and grew in fruit but not dairy matrices (data not shown). 

Moreover, several insertion sequences (ISs) were annotated with EggNOG and the ISfinder web tool. A total of 13 and 25 ISs were predicted in different loci on the UTNGt2 and UTNGt21A genomes grouped in eight families. The types of IS and their distribution in both genomes are depicted in Figure S2. Early genome comparison analysis between *L. casei* ATCC334 and other sequenced lactobacilli revealed a relatively high number of IS elements and carbohydrate-related genes [40]. The UTNGt2 genome harbors two distinct transposases—IS256 and ISNCY—while UTNGt21A showed three different transposases, IS3_ssgr_IS3, IS1182, and IS5_ssgr_IS1031 (identified members of the IS family according to ISsaga annotation). These genomic datasets are likely to provide answers about strains’ adaptation and ecological strength, as well as their role in distinct environments. 

### 3.3. Detection of Variants (Insertions, Deletions, SNPs) and Their Impact on the Genomic Architecture

**Figure 4:**
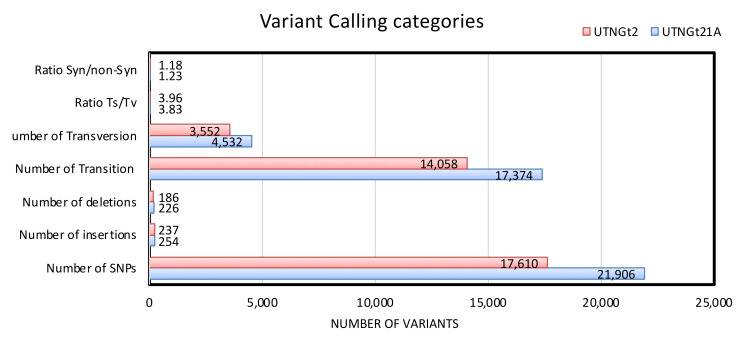
Cumulative summary of variant calling categories detected in the UTNGt21A and UTNGt2 genomes. Legend—Library name: sample name; Number of SNPs: number of SNPs in sample; Number of insertions: number of insertions in sample; Number of deletions: number of deletions in sample; Number of Transition (Ts): number of transitions in sample; Number of Transversion (Tv): number of transversions in sample; Syn: number of synonymous variants in sample; non-syn: number of non-synonymous variants in sample.

The gene variants (SNPs and short indels) were identified by analyzing the information taken from aligned reads. The variants were classified by each chromosome or scaffold, and the information of the location was marked. Although similar reads were mapped (10,191,789–10,428,975) for both strains, 22,386 and 18,033 variants were detected in the UTNGt21A and UTNGt2 genomes, respectively. Table S2 discloses the base changes for every SNP relative to the reference strain. The summary of the variant calling for each sample is depicted in Figure 4. Moreover, the numbers of transitions (Ts) and transversions (Tv), along with the Ts/Tv ratio, were calculated according to the base change count. Although there are twice as many possible transversions, transitions are more common than transversions due to differences in structural characteristics. The Ts/Tv ratio between homologous strands of DNA is generally ~2.0, but it is typically elevated in coding regions where transversions are more likely to change the underlying amino acid, thus possibly leading to a fatal mutation in the translated protein [41]. For UTNGt21A, the ratio percentage of Ts/Tv was 3.96 and 3.82 for UTNGt2, while the synonymous variants/non-synonymous variants ratio was 1.18 and 1.23 for UTNGt21A and UTNGt2, respectively. Generally, transitions are less likely to result in amino acid substitutions, thus remaining as "silent substitutions" in populations as SNPs, while transversions are more likely to cause amino acid sequence changes. The numbers of SNPs, Ts, and Tv were greater within the UTNGt21A genome. A total of 21,906 SNPs were detected in the UTNGt21A genome, while 17,610 were detected in the UTNGt2 genome. We speculated that the higher number of SNPs might be related to the larger genome of the UTNGt21A strain (3.82 Mpb). Table 2 depicts the number of variants and a brief description of the top 10 types of annotations. The synonymous variant acquires a maximum ratio of 71.51 and 72.01 within the UTNGt21A and UTNGt2 genomes, respectively, which is indicative of a sequence variant where there is no resulting change to the encoded amino acid.

A total of 32 (0.19%) and 21 (0.15%) stop-gained variants of genes involved in the defense mechanism were annotated within the UTNGt21A and UTNGt2 genomes, respectively. A stop_lost (c.553T>G; p.Ter185Gluext) and stop_gained (c.150C>A; p.Cys50) mutation trigger the *tnp*R1 locus within both native strains. These genes encode a serine recombinase protein (resolvase) that catalyzes the site-specific recombination of the transposon and regulates its frequency of transposition. A genomic evolutionary study of some *Lactococcus* strains indicated the adaptation of the strains to the milk culture medium because of the loss of the mobile elements [7]. In addition, the UTNGt2 genome harbors six noncoding_transcript_exon_variant counts detected in the lp_rRNA locus (transcribing 16S rRNA). Although the bacteria harbor far fewer noncoding exon variants, their function remains unknown. According to early research, noncoding exons are functionally interchangeable, with alternative splicing generating a larger number of potential regulatory RNAs and an enormous transcriptional repository for gene evolution [42]. Moreover, a stop_lost mutation was detected within the ISP2_1 gene (c.1653A>C; p.Ter551Tyrext) of the UTNGt21A genome. This protein showed a specific hit (E-value: 8.17 × 10^−16^) on COG3666 transposase and the transposase DDE domain (pfam01609) (E-value: 1.58 × 10^−16^) protein required for efficient DNA transposition [43]. These transposases were detected in several *L. plantarum* strains, indicating the complexity of the genome and genetic variation of these species [43]. However, these variants might contribute to the overall genomic plasticity and genetic variability, offering occasional mutations to cope with environmental changes and adapt to novel environmental niches. 

Additionally, 61 and 33 frameshift_variants were annotated within the UTNGt21A and UTNGt2 genomes, respectively. Among them, some strain-specific variants were detected within genes involved in carbohydrate metabolism and adherence-associated mucus-binding proteins. For example, a deletion was detected within the *pst11A* (c.13_16delAAAA; p.Lys5fs), *dprA* (c.134delC; p.His46fs), and *aapA* (c.1865delA; p.Gln622fs) genes in the UTNGt21A genome only. The PTS system is essential for sugar metabolism [3], while DprA protects against incoming foreign DNA [44]. AapA is an adherence-associated mucus-binding protein, which contributes to the persistence of bacteria in the human gut, thus exerting probiotic effects [45]. The frameshift, stop_gained, stop_lost, and splice_region variants cause gene expression changes, and may affect the functional properties of the encoded protein when the mutation is located within the coding region. SnpEff reports putative variant impact to make it easier and faster to categorize and prioritize variants. However, the impact categories must be used with care, as they were created only to help simplify the filtering process. Nevertheless, we cannot predict whether a high, moderate, modifier, or lower impact variant is generating a phenotype of interest; further *in vitro* analyses are required to demonstrate this statement. 

### 3.4. BGC Orgnization and Detection of Gene Variants Might Explain the Inhibitory Strength of the Native Strains

**Figure 5:**
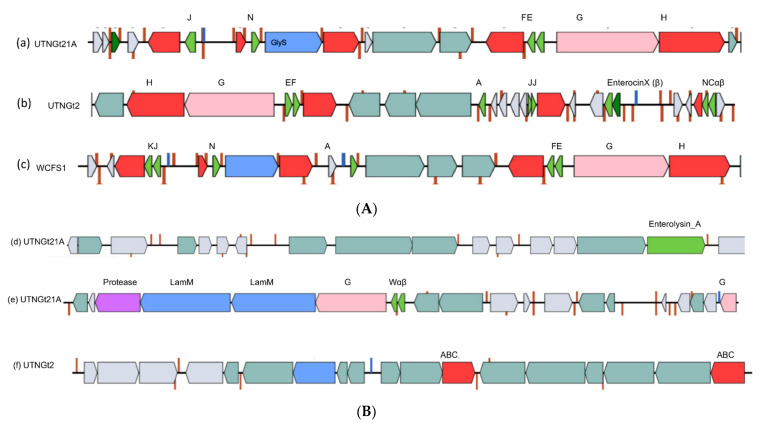
(**A**) Genetic organization of the *pln* loci of (a) UTNGt21A; (b) UTNGt2; and (c) WCFS1. (**B**) Additional bacteriocin cluster genes detected on (d, e) UTNGt21A and (f) UTNGt2. Legend—*A: plnA; J: pln J; N: plnN*; K: *plnK; E: plnE; F: plnF; NCαβ: NCα* and *NCβ; enterocinX (β): enterocin X* (chain beta); GlyS: glycotransferase family 2 protein (PlnO), LamM: lantibiotic mersacidin-modifying enzyme; Wαβ: Plantaricin_W (alpha), Plantaricin W (beta); 183.2; Plantaricin_W (beta); ABC: ABC-type bacteriocin transporter. Red blocks: immunity and transport; green arrow: core peptide.

The plantaricin genes are arranged in operons *plnABCD*, *plnEFI, plnJKLR, plnMNOP*, and *plnGHSTUVWX* [3]. Genome annotation analysis revealed the presence of a common two-peptide bacteriocin (plnEF) and several genes encoding for the ABC transport system. The genomic organization of the *pln* locus of the three strains is depicted in Figure 5A. Based on the EggNOG results, the *pln*A gene showing 100% identity with the reference gene (locus lp_0415) was annotated in the UTNGt2 genome [16], but not the UTNGt21A genome. Nevertheless, 12 *pln*A downstream variants (located at 3’of gene) were annotated in the UTNGt21A genome, while 20 upstream variants were detected in the UTNGt2 genome. The types of variants annotated, and the corresponding products, are shown in Table S3. In addition, a greater number of *pln*B, *pln*C, and *pln*D variants were annotated in the genome of UTNGt2, with the most abundant missense variant (15 counts) of the *pln*B gene. The impact of these mutations on gene product function or inhibitory capacity cannot be assessed by gene analysis. Nevertheless, the antimicrobial capacity of the UTNGt2 strain was demonstrated by different molecular assays [16]. Moreover, a frameshift variant (TGG insertion) was detected within the *pln*J gene of UTNGt21A (c.154_155insC; p.Ile53fs), but not UTNGt2 (Table S3). In addition, enterolysin_A, protease, plantaricin W (alpha and beta), and LanM were detected in the UTNGt21A genome only (Figure 5B). A putative enterolysin_A was previously detected in the genome of *Weissella cibaria* strain UTNGt21O [17], sharing the same ecological origin as the UTNGt21A strain. Moreover, multiple genome sequence alignment was performed with Jalview (version 2.10.1) [46], and the average distance was calculated based on the percentage of sequence similarity between enterolysin_A from UTNGt21A and the sequences from UTNGt21O and *Enterococcus faecalis* (NCBI accession no. AGG79281.1) strains (Figure S3A,B). However, the UTNGT21A and UTNGt21O showed 50% sequence identity; thus, we speculated that this bacteriocin could be acquired during a horizontal gene transfer between species inhabiting the same microenvironment, but additional analyses are needed in order to confirm this statement. No gene variants were detected, as the resequencing genome annotation was based on the available genomic information of the reference strain. Two hypothetical proteins of class IIb bacteriocin—a hypothetical protein encoding the plantaricin NC8 alpha-peptide precursor (97.87% identity to *L. plantarum* TBX52118.1) and the plantaricin NC8 beta-peptide precursor (100% identity to *L. plantarum* subsp. plantarum NC8)—were annotated within the UTNGt2 genome only. In addition, within contig 4 of UTNGt2, two ABC bacteriocin transporters were found (Figure 5B). Similarly, within the UTNGt21A genome, several species-specific lactococcin-G-processing and transport ATP-binding proteins were predicted. In a recent complementary inhibitory analysis, we evaluated the effects of the peptide extracts from both native strains on the whole-protein profile of *Staphylococcus aureus* ATCC1026. The results indicated a divergent protein pattern as a result of the effect of the peptide treatment on the release of several low- and high-mass proteins (Figure S4). These results are consistent with the findings of our previous study [47], suggesting that the overall inhibitory capacity depends on the peptide–protein extract mixture released in the extract, and is interconnected with the strains’ bacteriocin-encoding repertoire and gene variants, along with their distinct molecular mechanism of action. Decoding the antimicrobial capability via both in silico and in vitro analysis, as well as further coupling with ex vitro evaluation of the inhibitory action, will help to prospect their use as alternatives to conventional antibiotics. 

### 3.5. Gene Variants Might Play Important Roles in the Strains’ Adaptability to Different Stressors and Overall Probiotic Performance

The adaptation capabilities are species-specific and correlate with the genetic repertoire and gene variants of the target strain. The probiotic features of lactobacilli require the survival of the target strain under several stress conditions—including acidic environment, bile salt, and osmotic conditions—along with good viability that must be maintained during product manufacturing conditions, such as temperature and oxidative stress [48]. Moreover, the strains must meet the safety requirements [49]. However, as any changes in gene and protein expression might occur during severe conditions, the strains might possess several protectors’ molecules [50]. 

Survival under acidic conditions is one of the critical parameters of LAB strains if intended to be used as probiotic cells. The key regulator of intracellular pH is the F0F1-ATPase system [51,52]. The EggNOG annotation indicates the presence of eight genes belonging to the F0F1-ATPase system in both native strains and reference WCFS1 (Table 3). Analysis of NGS variant counts indicated that the UTNGt2 strain harbored more variants of the ATP synthase subunits alpha, beta, delta, gamma, epsilon chain, and subunit b, while UTNGt21A displayed variants in subunits alpha and beta only (Table S4). Both strains share the same SNP type of the *atp*A gene, with a conservative in-frame insertion (c.1501_1502insCCGCTG; p.Thr501_Ala502insAlaAla) and an upstream gene multi-nucleotide variant (TCCC). Genome resequencing indicated the presence of several SNPs (synonymous_variant) of the *lepA_1* gene (encoding for an elongation factor A)*,* with one missense_variant detected in UTNGt21A only (c.1354G>T; p.Val452Phe). Furthermore, four missense variants (c.82T>G; p.Ser28Ala; c.850A>G; p.Thr284Ala; c.856C>T; p.Arg286Trp; and c.1150G>A; p.Val384Met) and eight synonymous_variants within the *lepA_2* gene were detected in the UTNGt2 genome only (Table 3). It has been found that the elongation factors are involved in the attachment of bacteria to the human intestine and mucins [53]. How these mutations will affect the strains’ bacterial cell attachment requires further investigation. Nevertheless, we suggest that the fruit acidic microenvironment heritage and the genetic resistance machinery of these strains might support their further survival in low-pH habitats. 

A crucial parameter for the survival of the strain in the digestive tract is bile tolerance [54]. Early research indicates that the bile salt hydrolase gene *bsh* might be responsible for the tolerance of some *L. plantarum* strains to bile [55]. Nevertheless, there is no evidence of a direct connection between Bsh and bile resistance [54]. In the genomes of both native strains, no *bsh* genes were annotated. Instead, a conjugated bile acid hydrolase gene *cbh* (lp_3536) was detected along with three putative choloylglycine hydrolases (*yxel_1, yxel_2,* and *yxel_3*)—proteins involved in bile hydrolysis (Table 3). However, complementary in vitro analysis indicated that both native strains can adapt and maintain for 4 hours in 0.3% bile salt, with a slight increase in viability for UTNGt2 (Figure S5). 

The LAB strains are exposed to various temperatures, as the products that incorporate the cells must fulfill the safety requirements of the food sector [56]. Thus, several genes encoding proteins involved in heat shock stress resistance—such as 18kDa, heat shock protein, protein 15, and GrpE (methionine synthase)—were annotated with EggNOG within the genomes of both strains. Previous research indicates that by exposure to acidic pH (2.0) and incubation at 37 °C the GrpE and 30S ribosomal proteins were induced in some *L. plantarum* strains isolated from olive and corn silage [57]. Likewise, the chaperonins (GroEL) and cofactors (GroES) that play essential roles in promoting the correct folding and subsequent translocation of nascent polypeptides were annotated in both native strains (Table 3). Furthermore, two genes—*ccpA* and *ccpB*, encoding a catabolite control proteins A and B, respectively—were annotated in both native strains. The lack of these proteins affected the survival of the strain under heat [58].

Cold stress resistance is a determining factor of probiotic features [59]. Cold or freezing can diminish the cell viability considerably, inducing cell membrane damage and, consequently, altering the aromatic molecules [60]; thus, to overcome these harmful effects, the LAB genome is equipped with cold stress response genes encoding proteins such as CSPs [61]. Three cold stress proteins (*csp, cspL,* and *cspLA*) were annotated in both native strains. One SNP of the *csp* gene (c.144T>C; p.Thr48Thr) was found. In a recent complementary in vitro evaluation of the UTNGt2 cell viability changes during storage, under freezing (−80 °C) conditions, in a juice matrix, a decrease of 10% was detected in the samples stored for 12 months; nevertheless, the number of cells was greater (1.28 × 10^7^ CFU/mL) than the probiotic threshold value (1.0 × 10^6^ CFU/mL), suggesting high adaptability to cold stress (unpublished data). Similar results (5% viability decrease) were obtained after the storage of a milk-based product supplemented with UTNGt2 for 21 days under refrigeration—the remaining cells (2.09 × 10^7^ CFU/mL) were superior to the threshold limit, indicating high adaptability under cold conditions. A different performance was obtained with the UTNGt21A strain, where the cell viability decreased in milk but not in the fruit matrix (unpublished data). 

During food processing, the LAB strains must tolerate the osmotic changes that can affect the growth of the cells and their metabolism; therefore, they must overcome these changes by developing a molecular system against osmosis stress that includes two ABC transporters (*opu*ABCD, *cho*SQ) [57]. Early genomic characterization of the reference *L. plantarum* WCFS1 indicates that these systems are related to the biosynthesis and uptake of the osmoprotectants such as glycine, betaine, carnitine, and choline [9]. A divergent number of variants were annotated. Among them, two missense_variants of the *opu*B (c.136C>T; p.Pro46Ser) and *opu*C (c.567G>T; p.Lys189Asn) genes were detected in the UTNGt21A genome only (Table 3). An early study indicated that these molecules might protect against salt-induced cell injury [47]. Moreover, a superior number of mutations (19) were annotated within the genes (*mutS, mutY,* and *mutT*) encoding for proteins involved in the DNA mismatch repair mechanism of the UTNGt21A genome. In contrast, 10 mutations were detected within the UTNGt2 genome. Previous studies suggest that there is a correlation between the frequency of mutations within the proteins involved in the repair mechanisms and the adaptation of the strain to the dairy matrix [7]. As already mentioned above, at this point, we cannot predict whether these variants are producing a phenotype of interest. Further in vitro analysis might help to understand their achieved probiotic properties in the desired food matrix or their effectiveness as antimicrobial-producing strains. 

## 4. Conclusions

The identification of SNPs from the whole-genome resequencing is challenging in the context of the LAB strains’ adaptability to various niches and their further biotechnological performance. In this study, the genomic comparison of two native lactobacilli strains illustrates a distinct genetic repertoire and divergent gene variants that could be inherent to their microenvironment niches. Moreover, genomic comparison revealed that native lactobacilli harbored a plethora of genes participating in antimicrobial activity, with the UTNGt21A strain displaying a more complex bacteriocin cluster gene organization than UTNGt2. Pan-genome analysis disclosed the presence of various strain-specific genes involved in the defense mechanism and carbohydrate metabolism. The divergence between the functional gene cassettes detected within the GIs related to the carbohydrate metabolism might explain the strains’ response to designated niches. In addition, both native strains shared a common genetic pattern for stress tolerance; nevertheless, they were divergent in their types of gene variants, which might alter their adaptation and effectiveness if intended to be applied in different food matrices. The SNP counts and classes, the types of detected variants, and the impact of accumulated mutations on the strain phenotypes remain poorly understood, but can provide insights into the dynamics of the adaptation of LAB to different stressors or environments, as well as their probable technological applicability. Further in vitro analysis will direct the evaluation of probiotic technological properties and antimicrobial strength in different food matrices in order to better understand the impact of these variations on the overall strain utility.

## Figures and Tables

**Table 1:** Mapping data stats.

Library Name	Ref. Length	Mapped Sites (≥1x)	Total Reads	Mapped Reads	Mapped Bases	Mean Depth
UTNGt21A	3,348,624	3,028,007 (90.43%)	13,121,820	10,291,789 (78.43%)	941,582,288	281.18
UTNGt2	3,348,624	2,979,050 (88.96%)	11,733,026	10,428,975 (88.89%)	957,588,188	285.96

Note—Library name: sample name; Ref. Length: length of reference genome; Mapped Sites: length of mapped site; Total Reads: number of the total reads; Mapped Reads: number of reads mapped to the reference; Mapped Bases: number of bases in reads mapped to the reference; Mean Depth: average alignment depth.

**Table 2:** Cumulative types of variant annotation, descriptions, and impacts.

Type of Variant Annotation	Description	Impact *	Library Name			
			UTNGt21A		UTNGt2	
			Count	Ratio (%)	Count	Ratio (%)
synonymous_variant	Variant causes a codon that produces the same amino acid (e.g., Ttg/Ctg, L/L)	Low	12,350	71.51	9772	72.01
missense_variant	Variant causes a codon that produces a different amino acid (e.g., Tgg/Cgg, W/R)	Moderate	4740	27.45	3664	27
frameshift_variant	Insertion or deletion causes a frame shift (e.g., an indel’s size is not a multiple of 3).	High	61	0.35	33	0.24
stop_gained	Variant causes a STOP codon (e.g., Cag/Tag, Q/*)	High	32	0.19	21	0.16
splice_region and stop_retained_variant	A sequence variant in which a change has occurred within the region of the splice site, either within 1–3 bases of the exon or 3–8 bases of the intron/Variant causes stop codon to be mutated into another stop codon (the new codon produces a different AA). (e.g., Atg/Ctg, M/L (ATG and CTG can be START codons))	Low	18	0.1	22	0.15
conservative_inframe_deletion	One or many codons are deleted (e.g., a deletion multiple of three at a codon boundary).	Moderate	15	0.09	10	0.15
disruptive_inframe_insertion	One or many codons are inserted (e.g., an insertion multiple of three at a codon boundary).	Moderate	12	0.07	9	0.08
stop_lost and splice_region_variant	Variant causes stop codon to be mutated into a non-stop codon (e.g., Tga/Cga, */R)/A sequence variant in which a change has occurred within the region of the splice site, either within 1–3 bases of the exon or 3–8 bases of the intron.	High	9	0.05	5	0.07
disruptive_inframe_deletion	One codon is changed and one or many codons are inserted (e.g., an insert of a multiple of three in size, not at a codon boundary).	Modifier	6	0.03	6	0.04
conservative_inframe_insertion	Inversion of a large chromosome segment (over 1%, or 1,000,000 bases).	Moderate	6	0.03	6	0.04
non_coding_transcript_exon_variant	Region that does not code for any protein or does not carry genetic code.	Low	0	0	6	0.04

Note: * Impact—High: the variant is assumed to have a high (disruptive) impact on the protein, probably causing protein truncation, loss of function, or triggering nonsense-mediated decay; Moderate: a non-disruptive variant that might change protein effectiveness; Low: assumed to be mostly harmless or unlikely to change protein behavior; Modifier: usually noncoding variants or variants affecting noncoding genes, where predictions are difficult or there is no evidence of impact.

**Table 3:** List of genes and annotated variants associated with bacterial adaptation to different stressors in specific microenvironments.

Stress Factor	Gene (locus WCFS1)	% Identity (EggNOG Annotation)/No. of Variants Relative to the REFERENCE WCFS1				
		Protein Product	UTNGt21A		UTNGt2	
pH	*atpC* (lp_2363)	ATP synthase epsilon chain	67.60	(-)	67.60	1
	*atpD* (lp_2364)	ATP synthase subunit beta	84.79	2	84.79	1
	*atpG* (lp_2365)	ATP synthase gamma chain	64.19	(-)	64.19	2
	*atpA* (lp_2366)	ATP synthase subunit alpha	81.34	3	81.34	2
	*atpH* (lp_2367)	ATP synthase subunit delta	45.55	(-)	45.55	1
	*atpF* (lp_2368)	ATP synthase subunit b	57.64	(-)	57.64	1
	*atpE* (lp_2369)	ATP synthase subunit c	82.69	(-)	82.69	(-)
	*atpB* (lp_2370)	ATP synthase subunit a	54.85	(-)	54.85	(-)
	*lepA_1* (lp_2015)	Elongation factor 4	56.47	3	82.75	5
	*lepA_2* (lp_3120)	Elongation factor 4	82.75	(-)	56.63	12
Bile salt hydrolase	*yxeI_1*	Putative protein YxeI (Choloylglycine hydrolase)	42.98	(-)	42.98	(-)
	*yxeI_2*	Putative protein YxeI (Choloylglycine hydrolase)	40.54	(-)	34.85	(-)
	*yxeI_3*	Putative protein YxeI (Choloylglycine hydrolase)	34.85	(-)	40.55	(-)
	*cbh* (lp_3536)	Conjugated bile acid hydrolase	67.28	(-)	67.28	(-)
Temperature	*hsp2* (lp_2668)	18 kDa heat shock protein	44.96	3	42.05	3
	*hrcA* (lp_2029)	Heat-inducible transcription repressor HrcA	58.90	(-)	58.90	(-)
	*grpE* (lp_2028)	Protein GrpE	58.89	(-)	58.89	1
	*dnaK* (lp_2027)	Chaperone protein DnaK	84.33	(-)	84.33	3
	*dnaJ* (lp_2026)	Chaperone protein DnaJ	71.12	3	71.12	3
	*Gt21A_00947* *Gt21A_01250* *Gt2_02817*	18 kDa heat shock protein	44.9633.82	(-)	44.96	(-)
	*hslR*	Heat shock protein 15	70.79	(-)	71.91	(-)
	*groL* (lp_0728)	60 kDa chaperonin	84.89	2	84.89	2
	*groS* (lp_0727)	10 kDa chaperonin	69.14	(-)	69.14	1
	*hslO* (lp_0548)	33 kDa chaperonin	69.61	2	69.61	(-)
	*hsp 1* (lp_0129)	Hypothetical small heat shock protein	45.28	1	45.28	3
	*ccpA_1*	Catabolite control protein A	49.33	3	49.33	4
	*ccpA_2*	Catabolite control protein A	44.09		44.09	
	*ccpA_3*	Catabolite control protein A	65.76		65.76	
	*ccpA_4*	Catabolite control protein A	(-)		44.14	
	*ccpB*	Catabolite control protein B	44.92	1	47.60	2
	*cspP* (lp_1160)	Cold shock protein 1	78.78	1	78.78	1
	*cspL* (lp_0031)	Cold shock protein 2	81.81	(-)	81.81	(-)
	*cspLA*	Cold shock-like protein CspLA	86.36	(-)	86.36	(-)
Osmosis	*opuCD* (lp_1610)	Carnitine transport permease protein OpuCD	73.15	2	73.17	2
	*opuCC* (lp_1609)	Glycine betaine/carnitine/choline-binding protein OpuCC	63.10	5	63.49	2
	*opuCB_1* (lp_1608)	Carnitine transport permease protein OpuCB	98.21	5	75.00	3
	*opuCA* (lp_1607)	Carnitine transport ATP-binding protein OpuCA	68.62	3	68.62	3
	*opuCB_2*	Carnitine transport permease protein OpuCB	75.00	(-)	(-)	(-)
	*choS* (lp_0367)	Glycine betaine/carnitine/choline-binding protein	73.14	6	73.14	24
	*choQ* (lp_0368)	Glycine betaine/carnitine/choline-binding protein	68.62	3	68.62	(-)

(-): No variant detected.

## Supplementary Materials

The following are available online at https://www.mdpi.com/article/10.3390/genes13030443/s1. Table S1: GIs detected within the (A) UTNGt21A and (B) UTNGt2 genomes; Table S2: Base change count for every SNP of (A) UTNGt21A and (B) UTNGt2; Table S3: Variant annotation results of putative plantaricin genes of native *L. plantarum* UTNGt21A and UTNGt2 strains; Table S4: Variant annotation results of genes related to strains’ adaptation to different stressors; Figure S1: (A) Circular and (B) linear representation of the whole-genome comparison of *L. plantarum* UTNGt21A, UTNGt2, and WCFS1; Figure S2: Distribution and type of IS elements annotated with EggNOG and the ISsaga web tool; Figure S3: (A) Pairwise protein sequence alignment of putative bacteriocin from UTNGT21A and enterolysin_A from UTNGt21O and *Enterococcus faecalis* (NCBI accession no. AGG79281.1), with Clustal WS (v.2.0) retrieved from Jalview; (B) average distance calculated based on percentage similarity between sequences; Figure S4: Different expression profiles of *S. aureus* ATCC1026 treated with different peptide extracts; Figure S5: The effect of 0.3% bile on strain viability.
