# Supplementary material for: Decoding the Gene Variants of Two Native Probiotic Lactiplantibacillus plantarum Strains through Whole-Genome Resequencing: Insights into Bacterial Adaptability to Stressors and Antimicrobial Strength
Source: Genes (Basel). 2022 Feb 28;13(3):443. doi: 10.3390/genes13030443 (PMC8953754; doi:10.3390/genes13030443)
Supplement: Supplementary file 1 [file genes-13-00443-s001.zip › Figure S1.pdf]

Figure S1. (A). Circular; (B). linear representation of the whole-genome comparison of *L. plantarum* UTNGt21A, UTNGt2 and WCFS1. The outer and inner lines represent the scale in kbp. Circle 1 (red): all genes of Gt21A; circle 2 (brown): all genes of Gt2; circle 3 (orange): all genes of WCFS1 Circle 4 (green): Pangenome of Gt21A, Gt2 and WCFS1. Circle 5 (black line): GC content. Circle 6 (violet): GC skew; Circle 7 (multicolored): clusters of orthologous groups. The black box denotes the Gt21A insertions compared to WCFS1. rrf: ribosome recycling factor; The map was generated by web-program Gview Server Version v3. (https://server.gview.ca).

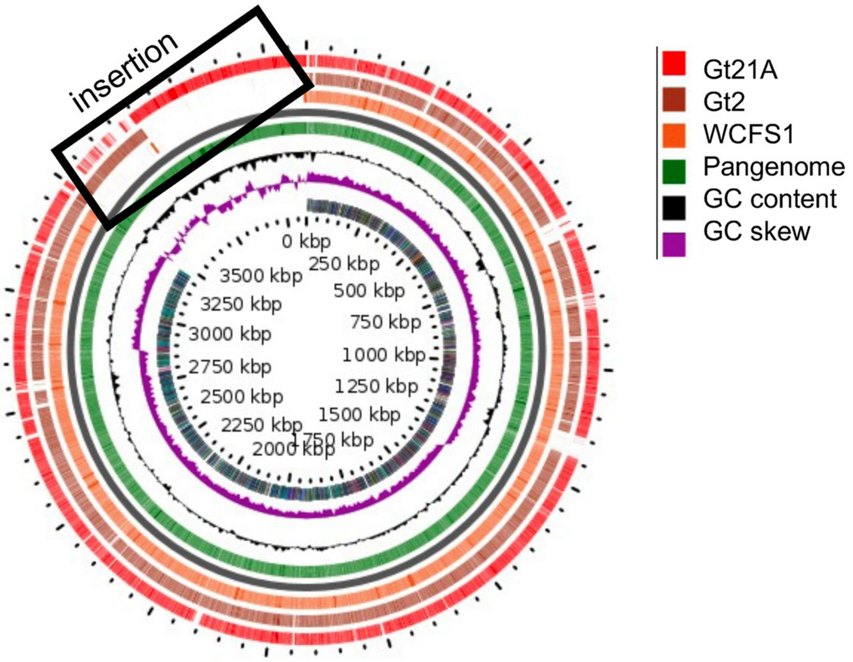

(A).

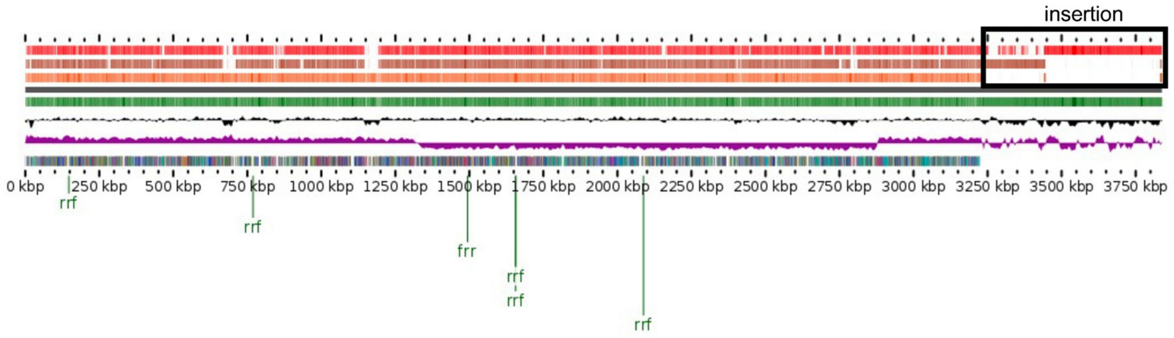

(B).
