# Supplementary material for: Decoding the Gene Variants of Two Native Probiotic Lactiplantibacillus plantarum Strains through Whole-Genome Resequencing: Insights into Bacterial Adaptability to Stressors and Antimicrobial Strength
Source: Genes (Basel). 2022 Feb 28;13(3):443. doi: 10.3390/genes13030443 (PMC8953754; doi:10.3390/genes13030443)

**Figure S2**. Distribution and type of IS elements annotated with EggNOG and ISsaga web tool. Inside ring depicted the IS elements predicte along the UTNGt21A genome; ourside ring displayed the IS elements predicted in the UTNGt2 genome.

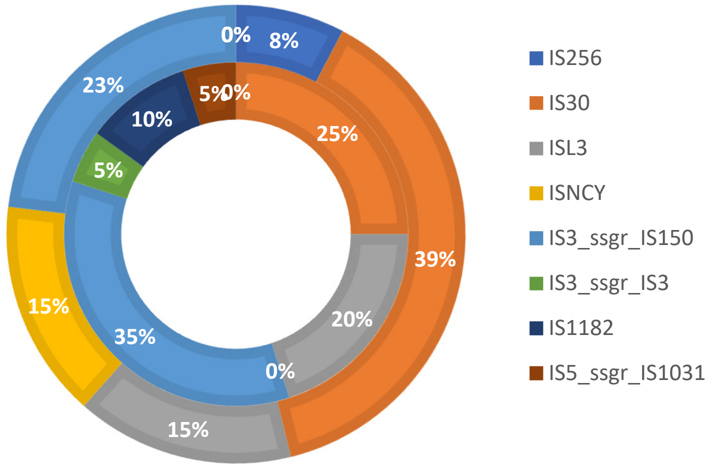

Supplement: Supplementary file 1 [file genes-13-00443-s001.zip › Figure S2.pdf]
