# Supplementary material for: Decoding the Gene Variants of Two Native Probiotic Lactiplantibacillus plantarum Strains through Whole-Genome Resequencing: Insights into Bacterial Adaptability to Stressors and Antimicrobial Strength
Source: Genes (Basel). 2022 Feb 28;13(3):443. doi: 10.3390/genes13030443 (PMC8953754; doi:10.3390/genes13030443)

**Figure S3.** A). Protein sequence pairwise alignment of putative bacteriocin from UTNGT21A and enterolysin_A from UTNGt21O and *Enterococcus faecalis* (NCBI accession no. AGG79281.1) with Clustal WS (v.2.0) retrieved from Jalview. *: indicate the similar amino acids. B). Average distance calculated based on percentage of similarity between sequences. The number on branch is the bootstrap value that indicates the extent of relatedness between two subjects.

```
UTNGt21A      MKNKWLALLFTLTTTLGLVFLGTTLKASANSVVRVTSDSATVYSGVDGTATRTLPINSAW   60
UTNGt21O      ------------------------------------------------------------   0
AGG79281.1    ------------------------------------------------------------   0

UTNGt21A      QYDQRVQGNGGAWWYRVSTNEWVKDTNVSESTQSAKNATGVVTVK---------NGKTAT   111
UTNGt21O      ---MNQADTATQIWNYLVGSGWSKTAVAAL-LGNMQSESGIIADRWQNDNVGNMSGGYGL   56
AGG79281.1    ------------------------------------------------------------   0

UTNGt21A      VRASLNGRAVGQTLASGTGWKYSQVAVDAWNFTWYHVSSTGWVS-----STDVTDNNSTK   166
UTNGt21O      VQWTPATKY--IDWAKANGLEYQNVYSQCKRLEWEVANNQQFSCPTMTFYQFKVSTDTPE   114
AGG79281.1    ----------------------------MKNILLSILGVLS-IVVSLAFSSYSVNAASNE   31
                                          .:      .              ..  : :

UTNGt21A      TGWPFARAYNGQYENGQQFGPNT---------------------------GTGRPNGF   197
UTNGt21O      ---NLAKIFITYYERPANPNQPERAQQARYWYDKLKNVDPKPKVKVVDWFNKHRGHITYS   171
AGG79281.1    WSWPLGKPYAGRYEEGQQFGNTA-------------------------FN--RG-GTYF   62
                  :.: :   **.  : .                                *  .

UTNGt21A      HDGFDFGSAIYGNSVFK------------------SIT-NGRVIYAQYYDS---VLKDVI   235
UTNGt21O      MDGSRIGTDGTADCSGSITIAVKESTGVPYDYVYNTVTLGGYLAKCGYTRILVGNVNGSN   231
AGG79281.1    HDGFDFGSAIY-------------GNGSVYAVH------DGKILYAGWDPVGGGSLGAFI   103
               **  :*:                                .* :  . :         :

UTNGt21A      VVQAPDGKQIMYQEFSSR-GD--NMYVHTGDTVTVGQSL---GYMSDSHLHVGVTGMNWQ   289
UTNGt21O      LNQMQD-EDIILLSEGTSMADSGGAGGHTGVISGGGKVITSTCYYTNGEANTAIQDLP--   288
AGG79281.1    VLQAGN-TNVIYQEFSRNVGD---IKVSTGQTVKKGQLIGK---FTSSHLHLGMTKKEWR   156
              : *  :  ::: . .   .*       **     *: :       :... : .:

UTNGt21A      VVQAPDGKQIMYQEFSSR-GD--NMYVHTGDTVTVGQSL---GYMSDSHLHVGVTGMNWQ   289
UTNGt21O      LNQMQD-EDIILLSEGTSMADSGGAGGHTGVISGGGKVITSTCYYTNGEANTAIQDLP--   288
AGG79281.1    VLQAGN-TNVIYQEFSRNVGD---IKVSTGQTVKKGQLIGK---FTSSHLHLGMTKKEWR   156
              : *  :  ::: . .   .*       **     *: :       :... : .:

UTNGt21A      SALHYAFTNNGTWINPITYIQNNLN-----------------------------------   314
UTNGt21O      -INANYLA--VNAFRYYEVW-RPSGTPNPDPTPVEFTANVYYGLRVLGGSWLGEVKNFNN   344
AGG79281.1    SAHSSWNKDDGTWFNPIPILQGGSTPTPPNPGPKNFTTNVRYGLRVLGGSWLPEVTNFNN   216
                        . :.

UTNGt21A      ------------------------------------------------------------   314
UTNGt21O      VDYDGFAGLPYHSHDLLYIKVDKGNLRYRTHSAQSGWLAWVDQGNPNDPVNGCAGNPGEA   404
AGG79281.1    T-NDGFAGYPNRQHDMLYIKVDKGQMKYRVHTAQSGWLPWVSKGDKSDTVNGAAGMPGQA   275

UTNGt21A      ------------------------------------------------------------   314
UTNGt21O      IDGVQIYYTTPAGQKLSQSYYRSQTTARSGWLGVCCDDGTSIAGFDGWAGMFGEPLDRLQ   464
AGG79281.1    IDGVQLNYITPKGEKLSQAYYRSQTTKRSGWLKVSADNG-SIPGLDSYAGIFGEPLDRLQ   334

UTNGt21A      ---------    314
UTNGt21O      IGISDKSPF    473
AGG79281.1    IGISQSNPF    343
```

A.

B.

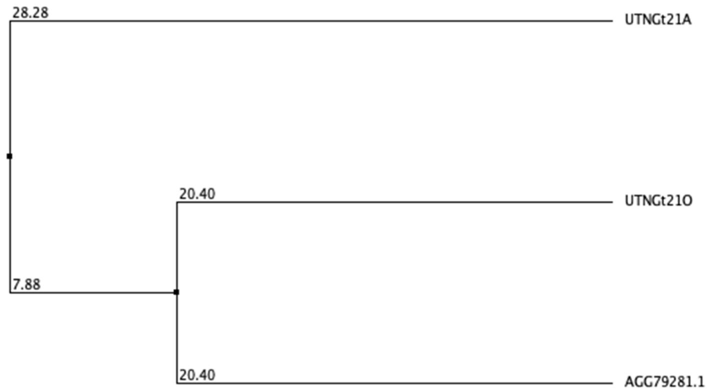

Supplement: Supplementary file 1 [file genes-13-00443-s001.zip › Figure S3.pdf]
