# Supplementary material for: Decoding the Gene Variants of Two Native Probiotic Lactiplantibacillus plantarum Strains through Whole-Genome Resequencing: Insights into Bacterial Adaptability to Stressors and Antimicrobial Strength
Source: Genes (Basel). 2022 Feb 28;13(3):443. doi: 10.3390/genes13030443 (PMC8953754; doi:10.3390/genes13030443)
Supplement: Supplementary file 1 [file genes-13-00443-s001.zip › Figure S4.pdf]

Figure S4. Different expression profiles of S. aureus ATCC1026 treated with different peptide extracts. Legend: lane 1, untreated S. aureus (control); lane 2, 3, 4: 1 X MIC of UTNGt21O, UTNGt2 and UTNGt21A peptide extracts at 24 h of incubation. M: molecular marker (Takara, Clearly Protein Ladder); arrows indicate different bands.

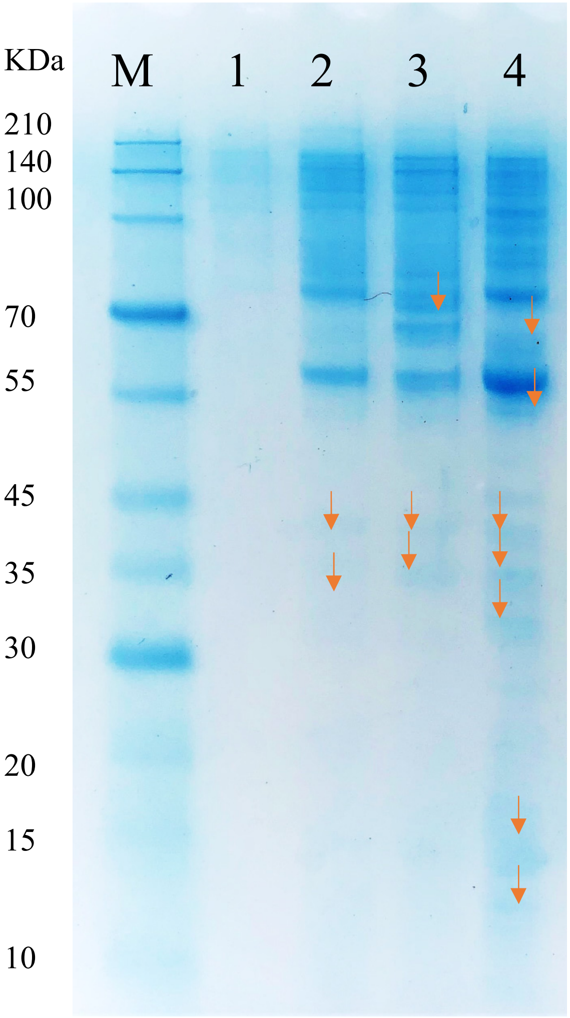

The following methodology was used: In brief, the effect of the peptide extracts on the whole target protein profile was analyzed using the SDS-PAGE method as previously described (Tenea and Hurtado, 2021). Samples containing S. aureus in BHI (Brain Heart Infusion, Merck Millipore, MA, USA) broth were incubated with 1 X-MIC of UTNGt21A and UTNGt2 peptide extracts at 37 °C for 24 h. Untreated cell with the peptide extract was used as a negative control. As positive control a peptide extract from Weissella cibaria UTNGt21O was used. The cell pellet was suspended in 1 X SDS-PAGE loading buffer, boiled for 5 min at 100 °C, and centrifuged at 300 x rpm. The supernatant of treated and untreated cells with the peptide extract was used in SDS-PAGE electrophoresis. The tricine-SDS-PAGE method using RunBlue Bis-Tris protein gels (12%) and Dual Cool Mini vertical PAGE/blotting Systems (Expedeon, Abcam, Cambridge, MA, USA) was used. The gel was stained with InstantBlue ready-to-use stain (Expedeon, Abcam, Cambridge, MA, USA) using a protocol recommended by the manufacturer.
