# Supplementary material for: Decoding the Gene Variants of Two Native Probiotic Lactiplantibacillus plantarum Strains through Whole-Genome Resequencing: Insights into Bacterial Adaptability to Stressors and Antimicrobial Strength
Source: Genes (Basel). 2022 Feb 28;13(3):443. doi: 10.3390/genes13030443 (PMC8953754; doi:10.3390/genes13030443)
Supplement: Supplementary file 1 [file genes-13-00443-s001.zip › Figure S5.pdf]

Figure S5. The effect of 0.3% bile on strain viability. Bars represent the means of three measurements ± standard deviation. *

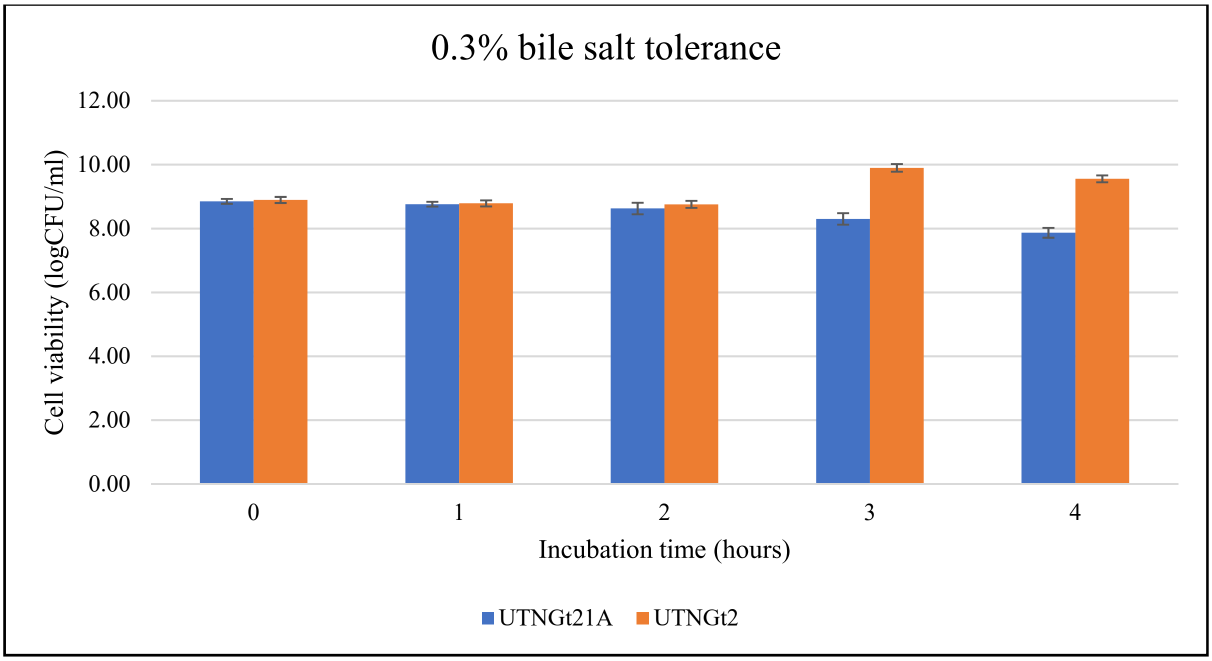

* The following methodology was applied: MRS broth media containing 0.3% of bile salts (#48305, Merck, USA) was inoculated with 500 µl of 24 h culture of each strain (O.D. = 0.0601-0.654); at different time intervals of 0, 1, 2, 3 and 4 hours, an aliquot of the culture was taken for the plate count. At the end of each incubation time, the number of viable cells was determined, and the data were expressed in log (CFU/ml).
