# Supplementary material for: Decoding the Gene Variants of Two Native Probiotic Lactiplantibacillus plantarum Strains through Whole-Genome Resequencing: Insights into Bacterial Adaptability to Stressors and Antimicrobial Strength
Source: Genes (Basel). 2022 Feb 28;13(3):443. doi: 10.3390/genes13030443 (PMC8953754; doi:10.3390/genes13030443)
Supplement: Supplementary file 1 [file genes-13-00443-s001.zip › Table S2.pdf]

Table S2. Base change count for every SNP of UTNGt21A (A) and UTNGt2 (B).

Library name		UTNGt21A	UTNGt2
**Ref**	**Alt**		
A	T	388	237
	G	4331	3520
	C	749	614
C	A	769	663
	T	4266	3472
	G	337	241
G	A	4396	3472
	T	801	593
	C	290	247
T	A	423	323
	G	775	598
	C	4381	3594

Legend.
Alt: DNA sequence of the sample
Ref: reference sequences regarding specific position
